# Editorial: Scalar Implicatures

**DOI:** 10.3389/fpsyg.2019.01767

**Published:** 2019-07-31

**Authors:** Anne Colette Reboul, Penka Stateva

**Affiliations:** ^1^UMR5304, Institut des Sciences Cognitives Marc Jeannerod, Bron, France; ^2^Center for Cognitive Science of Language, University of Nova Gorica, Nova Gorica, Slovenia

**Keywords:** scalar implicatures, variability, neo-Griceans, post-Griceans, grammatical approach

In 1975, Grice introduced the notion of implicature, arguing that it was more appropriate to account for a class of apparent lexical ambiguities through pragmatic processes than by multiplying lexical meanings (Modified Ockham's razor: *Do not multiply meanings beyond necessity*; Grice, 1975). His aim was to defend the idea that logical terms (*and, or, if… then*, quantifiers, etc.) do not have a meaning specific to their use in natural language. Rather, or so he argued, logical terms in natural language mean exactly what they mean in logic and their lexical meaning can be read off their logical truth tables. What gives the illusion that they acquire a different meaning in natural language is that their use in conversation frequently gives rise to implicatures. The following theoretical debate centered on how the pragmatic inferences necessary to access these implicatures were produced: neo-Griceans insisted on the specificity of scalar implicatures and on the importance of lexical scales (Horn, 1984; Levinson, 2000); post-Griceans rejected the idea that there was anything specific about scalar implicatures and emphasized the role of pragmatic processes (Sperber and Wilson, 1995; Noveck and Sperber, 2007).

For the past 20 years, experimental approaches have superseded purely theoretical ones, with mixed results. Paradigms using verification tasks on infelicitous sentences, with rate of pragmatic answers and reaction time as measures, have generally concluded in favor of the post-Gricean views (Bott and Noveck, 2004; Noveck and Reboul, 2008). However, some recent studies discuss additional factors affecting implicature processing and have introduced new paradigms which suggest a different conclusion (Katsos and Bishop, 2011; Breheny et al., 2013; Degen and Tanenhaus, 2015; Foppolo and Marelli, 2017; Bill et al.; Jasbi et al.; Sikos et al.). In addition, current research has shown that lexical scales may play a role in the process in keeping with neo-Gricean views (Doran et al., 2009; van Tiel et al., 2016; Gotzner et al.; Sun et al.). Furthermore, scales may vary in their potential to trigger pragmatic interpretations cross-linguistically. One possible explanation is that part of the variation may be due to the employment of different processes of pragmatic strengthening in different languages (Stateva et al.). Consequently, one might expect some more cases of cross-linguistic variation, notably among logical words (*or, if… then*, quantifiers, etc.).

This Frontiers topic is a collection of 12 contributions in experimental pragmatics focusing on different aspects of child and adult processing of implicatures, factors affecting their rate, relevance of testing paradigms, scale diversity, cross-linguistic differences, and variation in triggers.

A substantial part of the reported research examined various factors affecting the rates of pragmatic inferences, as well as their content. The role of prosody on restricting the relevant set of alternatives was given central attention in Chen et al. The study also investigated how context interacts with prosody. How prosodic stress on the scalar trigger influences pragmatic rates was also evaluated in one of the experiments reported in Bill et al. Two more studies investigate the effect of context on rates of pragmatic inferences. Yang et al's. article argues for a relation between individual cognitive resources, personality-based pragmatic abilities and language abilities, on the one hand, and sensitivity to context, on the other, which in turn, affects positively pragmatic rates. In their study, Sikos et al. manipulate social contexts to conclude that speaker's tolerance to pragmatic violations in the sense of Katsos and Bishop (2011) is affected in binary judgment task but not in graded judgments tasks. That study reveals another factor affecting rates of inferences: the number of response options in implicature comprehension studies. Whether the number of possible answers affects pragmatic rates is the main research question also in Jasbi et al. In its turn, this question raises important methodological considerations related to experimental designs in pragmatic studies and consequently the validity of the result interpretations. In line with Katsos and Bishop's (2011) evidence that a binary option task can mask children's ability to compute scalar implicatures, Jasbi et al. argue that a graded judgment design is more informative in evaluating rates of pragmatic inferences also in studies with adult speakers. However, designs involving a multiplicity of options necessitates careful effort in formulating the hypothesis that links the pragmatic inferences with the choice of provided answers. In addition to Jasbi et al's. discussion, this volume includes an article on the role of politeness in the comprehension of scalar implicatures which bears on the “linking hypothesis.” Mazzarella et al. distinguish between “comprehension” and “epistemic assessment” of communicated information. Their study reveals that it is possible to observe a discrepancy between rates of pragmatic answers and actually drawn inferences if the participants' evaluation of the truth of the potential inference is taken into consideration.

Scale diversity, as a major factor affecting pragmatic rates, and the source of the different potential of scalar triggers to incur inferences is discussed in Gotzner et al.; Sun et al.; Schaeken et al.; Stateva et al. and Gotzner et al. argue that scale structure related to a scalar item affects that item's potential to trigger scalar implicatures. In other words, properties (like gradability) of scale structures are a prerequisite for pragmatic strengthening not only by scalar implicatures but also by other kinds of inferences which can obscure each other's availability. Stateva et al. extend the topic of scale diversity and interaction of pragmatic enrichment processes to give it a cross-linguistic dimension. Schaeken et al. discuss scale diversity from the point of language acquisition. The study reveals different patterns of pragmatic rates in inferences related to quantitative vs. temporal scales. Sun et al. also explore potential factors responsible for the different implicature rates of scalar triggers and relate them to the susceptibility of different lexical items to local enrichment. This opens the door for an enlightening comparison between the grammatical theory of pragmatic enrichment and dual route theories. Evaluating the descriptive adequacy of different theories is also a topic of major interest in Bill et al. The article explores parallels and differences between scalar implicatures and presuppositions in patterns of processing. The results pave the way for further discussion in view of current proposals to subsume presuppositions under the umbrella of scalar inferences.

Buccola et al. offer an artificial word learning paradigm to examine competition which is at the core of pragmatic processes like computing scalar implicatures. The study demonstrates that symmetry among alternatives is another factor affecting the rate of inferences.

The corpus study reported Eiteljoerge et al. is one of the few available production studies of scalar implicatures. Its major contribution that children as young as 3 years of age can produce scalar inferences at rates comparable to their adult caregiver poses a curious puzzle in view of the acquisition delay observed in implicature comprehension studies (Noveck, 2001).

## Author Contributions

All authors listed have made a substantial, direct and intellectual contribution to the work, and approved it for publication.

### Conflict of Interest Statement

The authors declare that the research was conducted in the absence of any commercial or financial relationships that could be construed as a potential conflict of interest.
